# Machine Learning-Based Ensemble Recursive Feature Selection of Circulating miRNAs for Cancer Tumor Classification

**DOI:** 10.3390/cancers12071785

**Published:** 2020-07-03

**Authors:** Alejandro Lopez-Rincon, Lucero Mendoza-Maldonado, Marlet Martinez-Archundia, Alexander Schönhuth, Aletta D. Kraneveld, Johan Garssen, Alberto Tonda

**Affiliations:** 1 Division of Pharmacology, Utrecht Institute for Pharmaceutical Sciences, Faculty of Science, Utrecht University, Universiteitsweg 99, 3584 CG Utrecht, The Netherlands; a.d.kraneveld@uu.nl (A.D.K.); johan.garssen@danone.com (J.G.); 2 Nuevo Hospital Civil de Guadalajara “Dr. Juan I. Menchaca”, Salvador Quevedo y Zubieta 750, Independencia Oriente, Guadalajara C.P. 44340, Jalisco, Mexico; mendoza.lucero91@gmail.com; 3 Laboratorio de Modelado Molecular, Bioinformática y Diseno de farmacos, Seccion de Estudios de Posgrado e Investigación, Escuela Superior de Medicina, Instituto Politécnico Nacional, Mexico City 11340, Mexico; marletm8@gmail.com; 4 Life Sciences and Health, Centrum Wiskunde & Informatica, Science Park 123, 1098 XG Amsterdam, The Netherlands; a.schoenhuth@cwi.nl; 5 Genome Data Science, Faculty of Technology, Bielefeld University, Universitätsstraße 25, 33615 Bielefeld, Germany; 6 Global Centre of Excellence Immunology Danone Nutricia Research, Uppsalaan 12, 3584 CT Utrecht, The Netherlands; 7 UMR 518 MIA-Paris, INRAE, Université Paris-Saclay, 75013 Paris, France; alberto.tonda@inrae.fr

**Keywords:** miRNAs, TNBC, machine learning, feature selection, circulating

## Abstract

Circulating microRNAs (miRNA) are small noncoding RNA molecules that can be detected in bodily fluids without the need for major invasive procedures on patients. miRNAs have shown great promise as biomarkers for tumors to both assess their presence and to predict their type and subtype. Recently, thanks to the availability of miRNAs datasets, machine learning techniques have been successfully applied to tumor classification. The results, however, are difficult to assess and interpret by medical experts because the algorithms exploit information from thousands of miRNAs. In this work, we propose a novel technique that aims at reducing the necessary information to the smallest possible set of circulating miRNAs. The dimensionality reduction achieved reflects a very important first step in a potential, clinically actionable, circulating miRNA-based precision medicine pipeline. While it is currently under discussion whether this first step can be taken, we demonstrate here that it is possible to perform classification tasks by exploiting a recursive feature elimination procedure that integrates a heterogeneous ensemble of high-quality, state-of-the-art classifiers on circulating miRNAs. Heterogeneous ensembles can compensate inherent biases of classifiers by using different classification algorithms. Selecting features then further eliminates biases emerging from using data from different studies or batches, yielding more robust and reliable outcomes. The proposed approach is first tested on a tumor classification problem in order to separate 10 different types of cancer, with samples collected over 10 different clinical trials, and later is assessed on a cancer subtype classification task, with the aim to distinguish triple negative breast cancer from other subtypes of breast cancer. Overall, the presented methodology proves to be effective and compares favorably to other state-of-the-art feature selection methods.

## 1. Introduction

MicroRNAs (miRNAs) are noncoding RNA molecules of 18–25 nucleotides in length that regulate the expression of more than one third of human genes [1,2]. Since the discovery of the first miRNA in *Caenorhabditis elegans* [3], these molecules have been found in many organisms and tissue types. miRNAs have been shown to play an important role in cell biology, including differentiation, proliferation and apoptosis [4]. To date, there is evidence that miRNAs regulate different aspects of cancer development [5].

The biogenesis of miRNAs starts with a stem loop precursor created by RNA polymerase II, called primary precursor miRNA (pri-miRNA), that is cleaved by Drosa and DGCR8 proteins to obtain the precursor miRNAs (pre-miRNA) [6]. Finally, the pre-miRNA is cleaved by the Dicer/TRBP complex to create miRNA that represses or degrades the target mRNAs [7,8]. This machinery is altered in cancer cells, perturbing miRNA expression and accelerating the process of tumorigenesis. The discovery of cell-free circulating miRNAs in body fluids (blood, plasma, serum, urine, and cerebrospinal liquid) has put miRNAs in the focus of current research as promising cancer biomarkers [1,2,7,9,10,11,12]. Because the histological examination of tissues is an invasive and comparatively risky procedure, studying miRNAs in biological fluids offers a useful alternative for diagnosis, typing and management of cancer patients.

miRNA expression levels have proven to substantially vary relative to cell types. That makes it possible to use miRNAs to distinguish between cell types [13]. Furthermore, molecular signatures can be useful to differentiate between cancer types in general [14,15]. Another particularity is that these molecules are stable in extracellular environments: for example, they are resistant to pH and heat changes. Nowadays, the use of microarrays, real-time polymerase chain reaction (PCR) and next generation sequencing (NGS) technologies and the creation of databases give us the opportunity to study miRNAs as cancer biomarkers. Several studies have exploited the biomarker properties of miRNAs for cancer detection and classification, using machine learning techniques [16,17,18,19,20].

These works typically analyze thousands of different miRNAs, amounts that would make it impossible for medical experts to manually validate the results or to obtain novel insights. Furthermore, employing thousands of miRNAs in machine learning approaches translates into operating in feature spaces of thousands of dimensions, which nurtures the usual issues linked to the curse of dimensionality. Therefore, in addition to enhancing the interpretability of results, determining small, actionable subselections of features warrants approaches that are insensitive to biases emerging from batch effects (due to processing data from multiple studies, for example), from the use of sets of classifiers that vary in terms of their strengths and weaknesses or just from the nature of their technical foundations. Finding the smallest subset of circulating miRNAs that can identify the presence of cancer or the type of tumor is therefore of utmost practical importance.

In this work, we propose a new methodology to reduce the number of significant circulating miRNAs needed by machine learning techniques to detect and identify cancer types using 16 miRNA datasets from clinical trials. The technique relies on a heterogeneous ensemble of classifiers to provide more robust results than single algorithms or even homogeneous ensembles. The presented approach is first used to identify 10 different types of cancer, and then, in a second experiment, the same technique is applied to separate tumor subtypes in breast cancer. The methodology not only is proven to be effective but also compares favorably to current state-of-the-art techniques.

While a similar technique was presented in [21,22], the approach we propose features several improvements and important innovations that set it apart from previous contributions: (i) previous works did not select for circulating miRNAs, and thus, resulting signatures could not be easily measured in clinical practice; (ii) previous techniques needed extra parameters to be defined by the user (for example, a desired number of features), while the novel approach we propose does not require users to arbitrarily set values for thresholds; and (iii) finally, the amount of data used in the experimental verification greatly increased, getting a total of 16 gene expression omnibus (GEO) datasets.

## 2. Materials and Methods

First, we compiled a list of circulating miRNAs (mature sequence) based on 5 reviews of circulating miRNAs from cancer studies [1,2,23,24,25]. Next, from this list, we consider only the miRNAs that appear in blood, serum, urine, plasma and saliva. To narrow it further, we focus on the miRNAs that can be detected by Affymetrix platforms Affy-1 (GPL8786), Affy-2 (GPL14613) and Affy-3 (GPL16384). Our choice of restricting to datasets from Affymetrix platforms GPL8786, GPL14613 and GPL16384 has the aim of avoiding the known issue of miRNA expression levels being platform- and technology-dependent [26,27,28]. After this selection, a total of 253 miRNAs remain. The detailed list is included in Appendix A.

### 2.1. Feature Selection

As our objective is to select the most meaningful miRNAs to correctly classify the cancer types, we used a recursive ensemble feature selection algorithm where features in our datasets are *expression* values of different miRNAs. The idea behind recursive feature selection is to score each feature depending on its usefulness for the classification process, resorting to a classifier. Features with the lowest scores are then removed, and the process is iterated with the remaining features until the overall classification accuracy drops below a given threshold or when a user-defined number of features is reached. While this technique is effective, it still relies on a classification algorithm to score the features, and a single algorithm might be affected by bias when it assigns scores to the features. A way to reduce the bias is to exploit an ensemble of classification algorithms with different topologies, an idea that is proven to be effective for different problems [29,30,31].

For the ensemble, we selected 8 classifiers from the sci-kit learn toolbox [32] that all were proven to be effective for cancer classification using miRNAs [18] and that are able to score features according to their importance: Stochastic Gradient Descent (SGD), Support Vector Machine classifier (SVC), gradient boosting, random forest, logistic regression, passive aggressive classifier, ridge classifier and bagging. Parameters for each classifier, when different from the default, were taken from [18].

Different algorithms assess feature importance *differently*, as the scoring depends on the computational particularities of the algorithms. Bagging, gradient boosting and random forest use ensembles of classification trees. In these cases, we count the features that appear in the splits of the trees and rank them by frequency. For SVC, SGD, passive aggressive, logistic regression and ridge, the feature importance is given by the absolute value of the coefficients associated to each feature. Therefore, the ranking is based on the value of these coefficients.

As the ranking of each classifier has a different meaning, it is necessary to aggregate this information into an ensemble ranking. Each feature *f* is assigned a simple score $s_{f}=N_{t}/N_{c}$, where $N_{t}$ is the number of times that feature appears among the top *S* over all classifier instances, while $N_{c}$ is the number of classifier instances used. Each classification algorithm has 10 instances, produced by a 10-fold stratified cross-validation ($N_{c}=8\times 10=80$). The cross-validation is used to increase generality of the results. We selected a stratified cross-validation because it preserves the same ratio of samples for each class in the training and test. Next, the recursive feature algorithm will reduce the number of features *S* by 20% at each iteration. For our experiments, we decided to stop the procedure when the global average accuracy among all classifiers drops to less than 90%. The complete procedure is summarized by Algorithm 1.
**Algorithm 1:** Recursive ensemble feature selection.
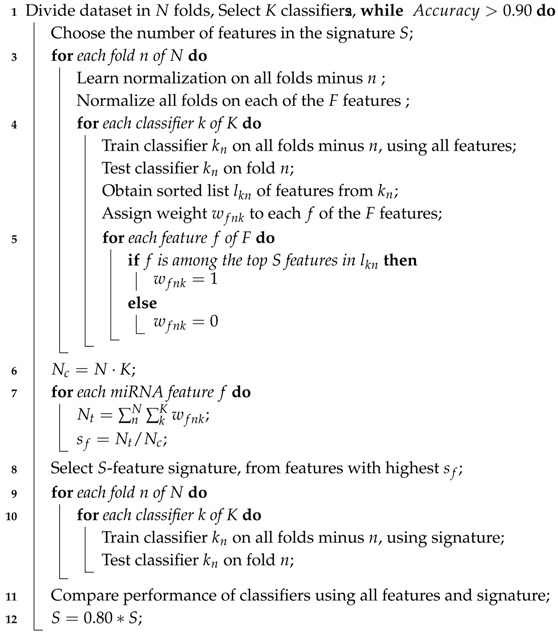


### 2.2. Cancer Type Classification

From the gene expression omnibus (GEO) repository [33], we selected 16 datasets for 10 different types of cancer based on clinical studies: Breast (BRCA), esophageal (ESCA), head and neck squamous cell (HNSC), liver hepatocellular (LIHC), prostate (PRCA), glioblastoma (GBM), colorectal (CRC), non-small-cell lung (NSCLC), gastric (GC) and ovarian (OVC), as summarized in Table 1. For each dataset, we downloaded the raw data and processed it using the function *Affyrma*() from the Matlab bioinformatics toolbox™. This function processes the probe intensity values using RMA background adjustment, quantile normalization and summarizing procedures and then outputs *expression* (nondimensional). The resulting aggregated dataset for our multi-class classification problem presents 845 samples, 253 features and 10 different tumor classes. Next, we applied Z-score normalization on the dataset to then run the feature selection algorithm in a 10-fold stratified cross-validation scheme.

Then, we compared our results against two current state-of-the-art feature selection methodologies: a homogeneous ensemble classifier exploiting variations of SVC [29] and a feature selection tool based on genetic algorithms, called GALGO [43]. Since each algorithm contains stochastic elements, we run each algorithm 10 times and keep the set of features with the best average accuracy.

The homogeneous ensemble uses several runs of SVC to rank the features by weight and reduces the number of features by a given percentage at each step. In this case, we used the same parameters as for Algorithm 1: 20% step reduction and 90% accuracy as stop parameters. In contrast, for GALGO to obtain a fair comparison, the requested number of features is set to the resulting number of features from the heterogeneous ensemble feature selection classifier.

Finally, we analyzed the genes targeted by the candidate miRNAs using miRNet [44]. The parameters for the miRNet analysis are target genes as main function with a 0.05 *betweenness* filter and pathway enrichment analysis using the Kyoto Encyclopedia of Genes and Genomes [45] (KEGG) and Gene Ontology-Biological Process [46] (GO:BP). Using a *betweenness* filter implies that the genes must be targeted by at least 2 miRNAs.

### 2.3. Triple-Negative Breast Cancer Classification

Cancer tumors are divided into tumor subtypes, which can be treated by different strategies depending on their classification. From the available data in the GEO repository, we were able to compile a dataset to assess the possibility of classifying tumor subtypes (luminal A, luminal B, HER2-enriched, triple-negative and normal [47]) in breast cancer (BRCA) using circulating miRNAs. Then, we selected datasets GSE86277, GSE86278, GSE86281 and GSE46823, which are BRCA studies with subtype information. From the BRCA subtypes, triple-negative has the worst prognosis, as it is resistant to hormone therapies [48]. For this reason, we set the labels of the resulting dataset to separate the triple-negative subtype from the rest. Although making an analysis of all the subtypes would have been more interesting, the unbalance in the subtype samples found in the original data makes it impossible; thus, more precisely, the resulting dataset has 139 triple-negative samples and only 44 from the rest of the subtypes, for a total of 183 samples, 253 features and 2 classes (triple-negative/Other).

Next, we applied the function *Affyrma* from the Matlab bioinformatics toolbox™. Then, we applied Z-score normalization on the dataset to run the feature selection algorithm in a 10-fold stratified cross-validation scheme. As in the previous experiment, the feature selection algorithm was set to identify the smallest miRNA subset sufficient to obtain a 90% accuracy. In addition, we compared our results with the 31-miRNA signature proposed by Romero et al. [35] to separate Triple-Negative Breast Cancer (TNBC) from other subtypes of BRCA using miRNA–mRNA integrative analysis in TNBC tumors based on the differential expressed transcripts. It is important to take into consideration that this 31-miRNA list considers noncirculating miRNAs that are not included in our method and could potentially access more information. Finally, we ran miRNet using the candidate miRNAs, as in Section 2.2.

## 3. Results

### 3.1. Cancer Type Classification

**Figure 1:**
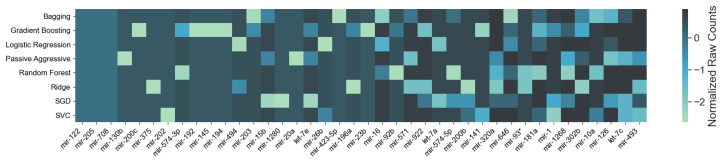
Feature importance by classifier: On the horizontal axis, the top features are reported, following their ensemble ranking. The intensity of the color in each square represents the frequency of appearance of that particular feature in the 10 instances of the same classifier produced by cross-validation; the darker the color, the more frequent the appearance of that feature among the most important. It is noticeable how different classifiers rank features differently. For this figure, we report the top 42 features only for visualization purposes.

**Figure 2:**
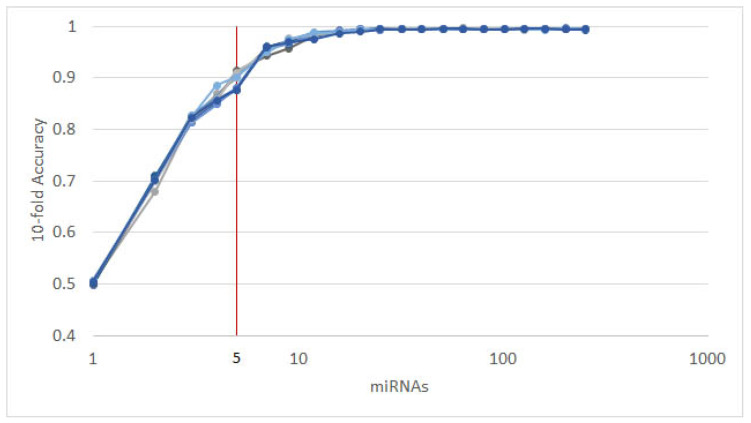
The results of 10 runs of the recursive ensemble feature selection for cancer type classification: The *x* axis cuts at 5 variables, where all runs cross the 90% average accuracy stop parameter (we computed the subsequent values as a reference).

As mentioned in Section 2.1, each of the classifiers ranks the features differently. When applied to the 253 circulating miRNAs, the top features obtained by each classifier appear as in Figure 1. From visual inspection, it is easy to observe how each classifier ranks the features differently. Nevertheless, the features where the classifiers concur are the most important. From the feature selection algorithm, we reduced the original 253 miRNA to 5, while maintaining an average accuracy of 90% over the selected classifiers (Figure 2).

**Figure 3:**
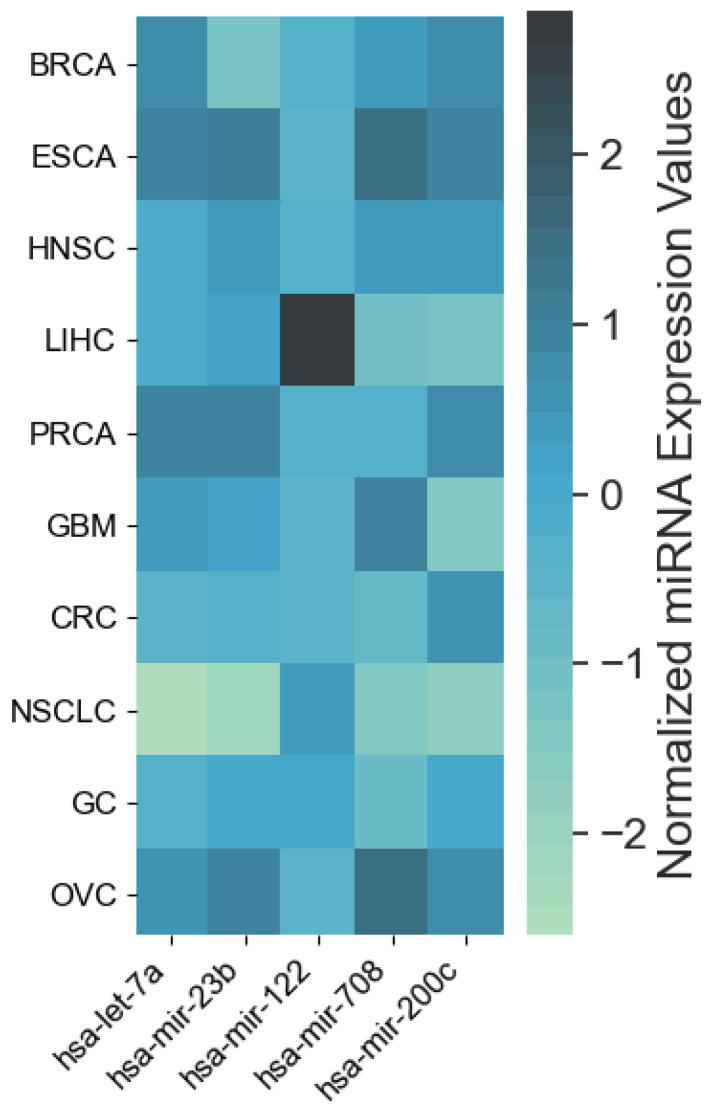
Heatmap of average expression levels by cancer type for the 5 miRNAs identified by the proposed approach. Cancer types: breat (BRCA); esophageal (ESCA); head and neck squamous cell (HNSC); liver hepatocelluar (LIHC); prostate (PRCA); gliobastoma (GBM); colorectal (CRC); non-small-cell lung (NSCLC); gastric (GC); ovarian (OVC).

**Figure 4:**
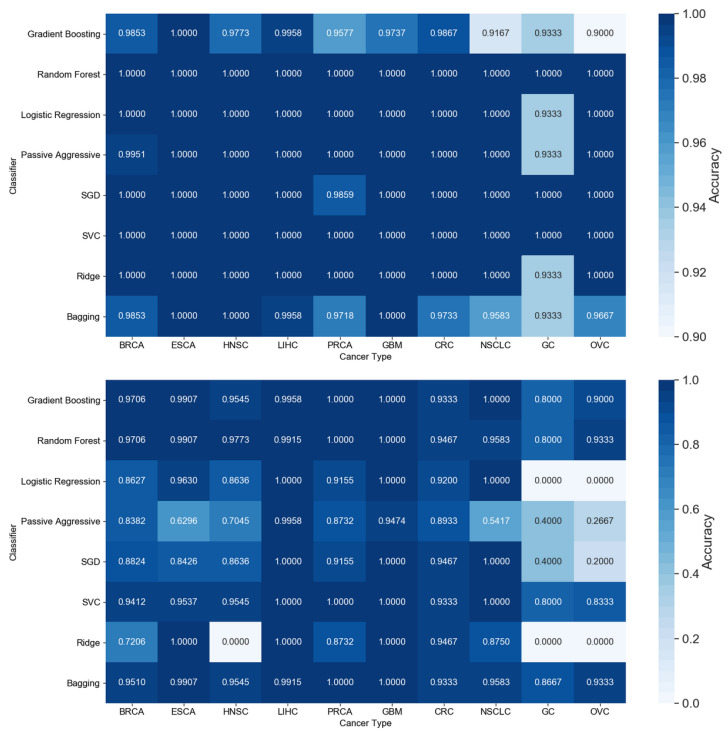
Comparison of accuracy by classifier and tumor type for all 253 features (top) and the 5 features identified by the proposed approach (bottom). Cancer types: breast (BRCA); esophageal (ESCA); head and neck squamous cell (HNSC); liver hepatocellular (LIHC); prostate (PRCA); gliobastoma (GBM); colorectal (CRC); non-small-cell lun g (NSCLC); gastric (GC); ovarian (OVC).

The resulting most significant 5 features uncovered by the presented algorithm are hsa-let-7a, hsa-miR-23b, hsa-miR-122, hsa-miR-708 and hsa-miR-200c, with seemingly different *expression* levels for each cancer type (Figure 3). For more detailed expression values by cancer type, see Appendix B. The classifiers gradient boosting, random forest, SVC and bagging seem to work in a satisfying way for all tumor types using only 5 miRNAs, whereas the rest have issues classifying the types of cancer HNSC, GC and OVC while having better performance when using the full 253 miRNAs, as shown in Figure 4. Interestingly, hsa-let-7 and hsa-miR-200c were also discovered by the homogeneous ensemble while GALGO’s performance seems to be less effective and has no miRNAs in common with our approach. From the comparison with GALGO and the homogeneous ensemble classifier with SVC, we can notice how the proposed heterogeneous ensemble classifier outperforms the other feature selection techniques in Table 2.

#### 3.1.1. Numerical Validation

**Figure 5:**
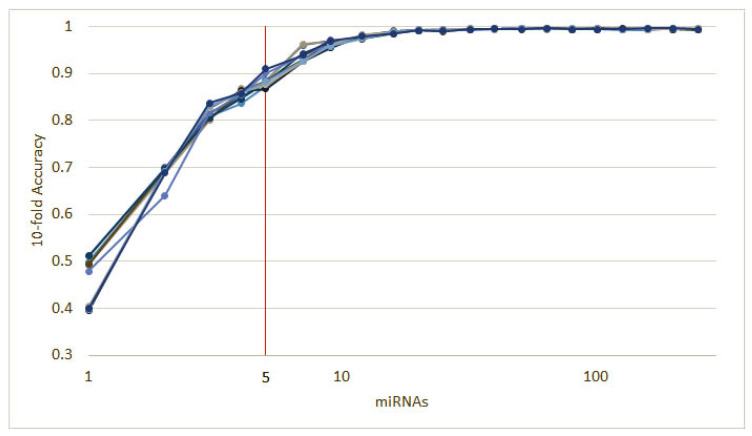
Ten runs of the heterogeneous ensemble recursive selection algorithm. From the 10 runs, the minimum number of necessary miRNA to have an accuracy above 90% is 5: hsa-let-7a, hsa-miR-23b, hsa-miR-122, hsa-miR-708, and hsa-miR-200c.

To further validate our methodology, we split the dataset described in Section 2.2 into a training (90%) and a validation (10%) subsets. Then, we ran 10 instances of the recursive ensemble feature selection algorithm with 90% of the data in a 10-fold cross-validation, which yields the curve in Figure 5.

Next, we selected the smallest signature that provided an accuracy of 90% or above, having as a result hsa-let-7a, hsa-mir-122, hsa-mir-200c, hsa-mir-708 and hsa-mir-23b, the same miRNAs identified in the previous experiment described in Section 3.1. Then, we tested this signature on the 10% subset, comparing against signatures identified by other approaches: homogeneous ensemble feature selection, GALGO, K-best univariate feature selection (using f-score) and 3 random selected subsets. In addition, we shuffled the test set’s labels to verify the proper workings of the classifiers (Table 3). Finally, we calculated the Matthews Correlation Coefficient (MCC) for all of the signatures and classifiers [49] (Table 4).

**Figure 6:**
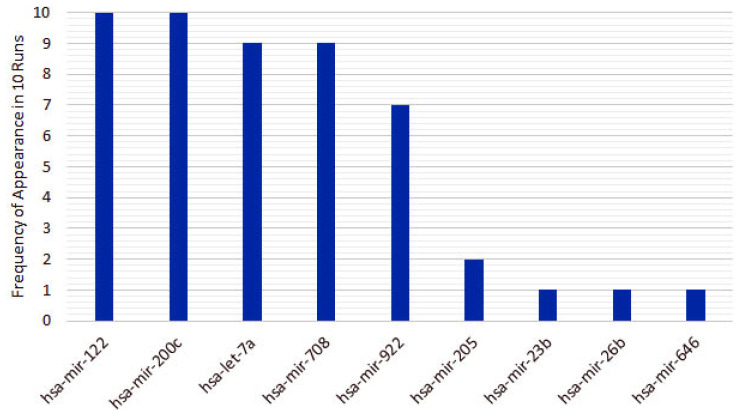
Ten recurrent features in the 5-feature signature for the heterogeneous ensemble feature selection algorithm.

**Figure 7:**
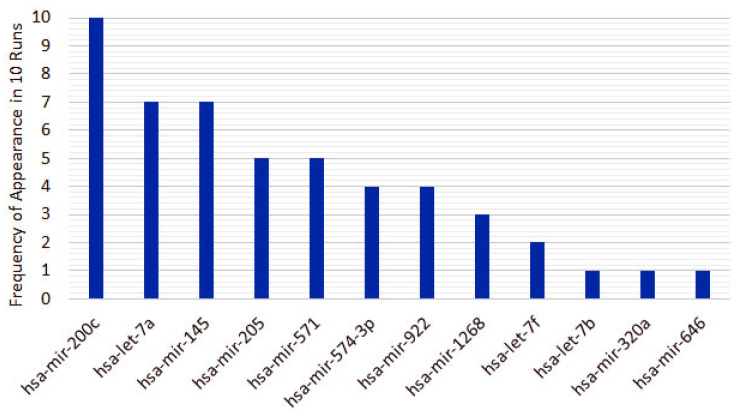
Twelve recurrent features in the 5-feature signature for the homogeneous ensemble feature selection algorithm.

**Figure 8:**
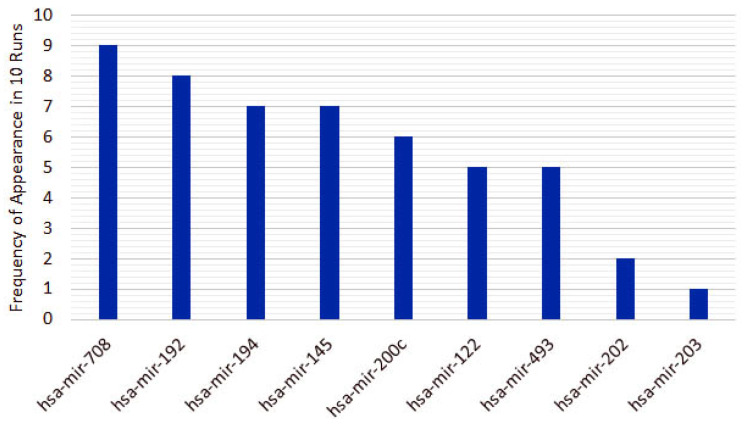
Nine recurrent features in the 5-feature signature for the GALGO.

From the 10 instances, we then measured the frequency of appearance of miRNAs in the top 5 features for each run. From the original 253 features, only 10 features appear in the top 5 for the heterogeneous recursive ensemble feature selection algorithm, with the frequencies presented in Figure 6. We repeated the same procedure for 10 runs of the homogeneous ensemble feature selection algorithm (feature frequency presented in Figure 7) and GALGO (feature frequency presented in Figure 8). The variability of the output signature for each algorithm reflected that the average and standard deviations for accuracy and MCC for the proposed heterogeneous recursive ensemble feature selection algorithm are better than the homogeneous recursive ensemble feature selection algorithm and GALGO (see Table 5).

#### 3.1.2. Pathway Analysis

**Figure 9:**
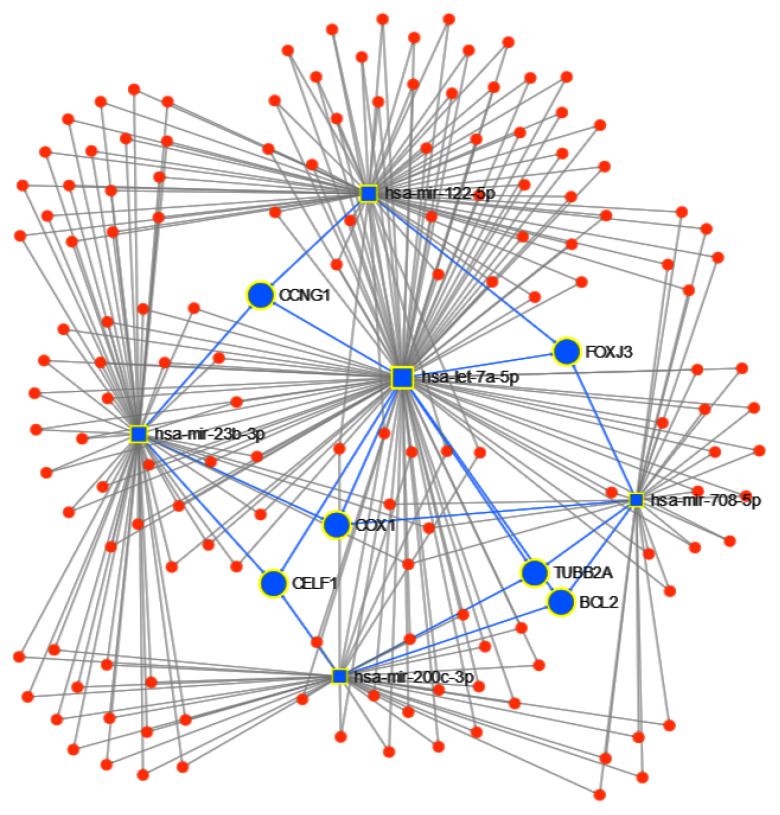
miRNET targeted genes analysis, showing genes targeted by at least 3 of the 5 miRNAs to classify cancer type: BCL2, CCNG1, COX1, TUBB2A, CELF1 and FOXJ3.

Next, using the 5 candidate miRNAs identified by the proposed approach to separate the tumor type, we ran miRNet to identify the targeted genes, obtaining a total of 1732 genes. After we apply a 0.05 *betweenness* filter, we reduced the list to 156 genes. From these genes, BCL2, CCNG1, COX1, TUBB2A, CELF1 and FOXJ3 are targeted by at least 3 of the 5 miRNAs (Figure 9). Finally, using the function explorer of miRNet, we performed a functional enrichment analysis with a hypergeometric test of the genes from the KEGG database and GO:BP. In Table 6 and Table 7, we show the results of the top 10 functional enrichment analyses for KEGG and GO:BP respectively. The first result in KEGG is the P53 signaling pathway. The P53 protein is a tumor suppressor protein, and it is involved in several anticancer mechanisms [50]. In the GO:BP database, the first result is the cellular response to stress, with 44 of the genes in the pathway; cellular stress is a component of the P53-mediated tumor suppression [51].

### 3.2. Triple-Negative Breast Cancer Classification

**Figure 10:**
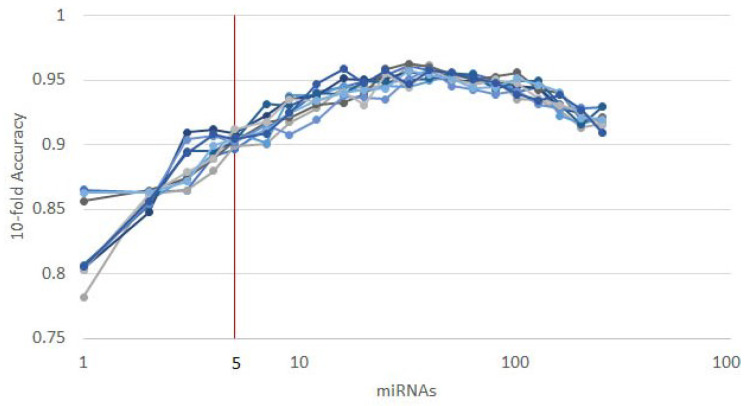
Results of 10 runs of the recursive ensemble feature selection for the TNBC discrimination example: The *x* axis cuts at 5 variables, which is where most evaluations cross the average 0.90 accuracy stop parameter.

**Figure 11:**
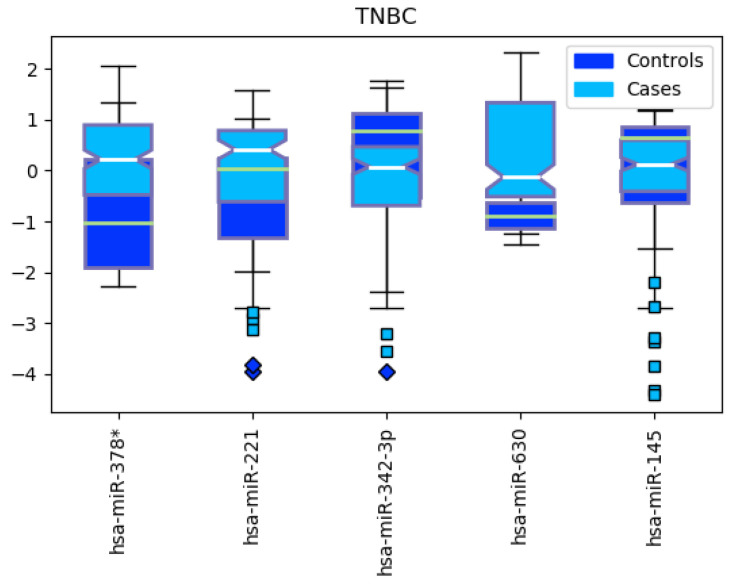
Boxplot for the *expression* levels between Triple Negative Breast Cancer (TNBC, cases) and other subtypes (controls).

We ran the heterogeneous ensemble algorithm 10 times, identifying 5 meaningful miRNA features for separating triple-negative BRCA from the other subtypes (Figure 10). The resulting miRNAs are hsa-miR-378*, hsa-miR-221, hsa-miR-342-3p, hsa-miR-630 and hsa-miR-145. The corresponding expression levels for the identified miRNAs in TNBC and non-TNBC are reported in Figure 11.

**Figure 12:**
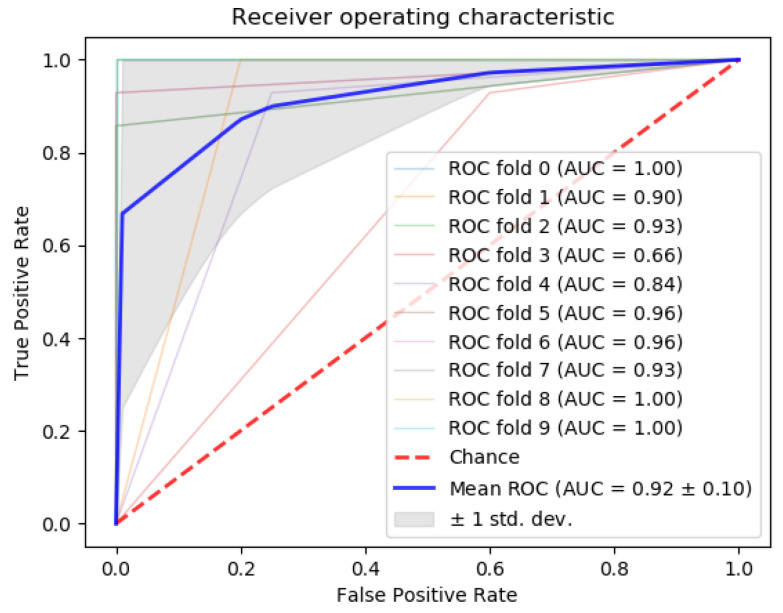
ROC curve using the gradient boosting classifier to separate Triple Negative Breast Cancer (TNBC) from the rest of the breast cancer subtypes.

Next, we compared the accuracy between classifiers using all 253 miRNAs in the dataset, our 5-miRNA signature, and the 31-miRNA signature proposed by Romero et al. for distinguishing TNBC from other cancers (Table 8). From the results, our algorithm outperforms the 31-miRNA signature. In addition, the area under the curve (AUC) of the results (Figure 12) calculated with the gradient boosting classifier is above 90%. This is considered *outstanding* results following the guidelines in [52,53] for clinical use of algorithmic methodologies.

**Figure 13:**
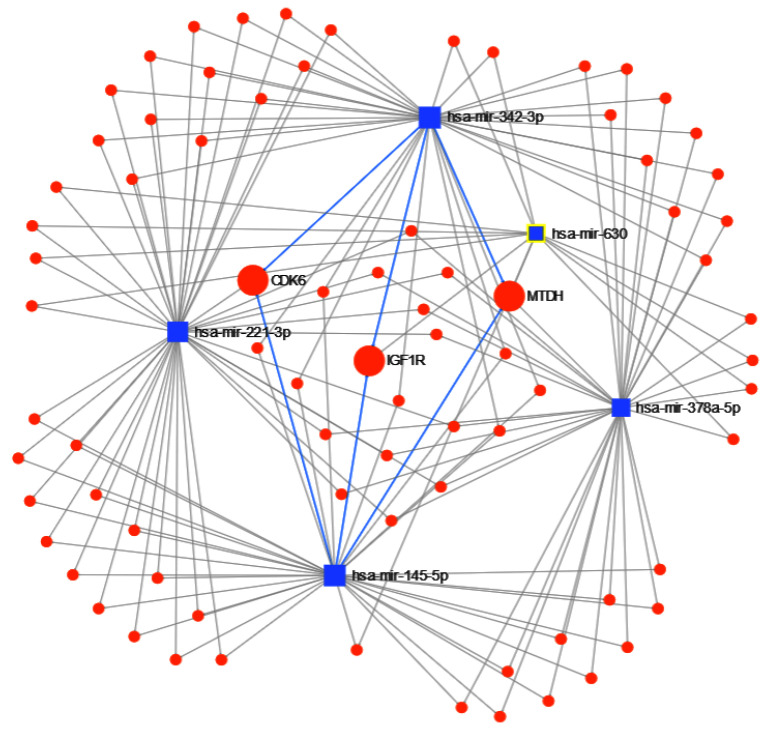
miRNET targeted genes analysis, showing genes targeted by at least 3 of the 5 miRNAs to separate Triple Negative Breast Cancer (TNBC) from other breast cancer subtypes: metastasis gene metadherin-positive (MTDH), type 1 insulin-like growth factor receptor-positive (IGF1R) and cyclin-dependent kinase 6-positive (CDK6).

Finally, the results of miRNet found 1294 genes targeted by the 5 miRNAs, with 79 having at least 2 miRNAs in common. From those 79, metastasis gene metadherin-positive (MTDH) is targeted by 4 miRNAs, while type 1 insulin-like growth factor receptor-positive (IGF1R) and cyclin-dependent kinase 6-positive (CDK6) are targeted by 3; see Figure 13. From the enrichment analysis, the most important functional pathway in the KEGG database (Table 9) is the *p53 signaling pathway* (the same identified in the previous experiments for separating cancer types), and in GO:BP (Table 10), the *negative regulation of cell proliferation*, with 12 of the 79 genes followed by *regulation of cell proliferation* and just *cell proliferation*. These results show an important involvement of cell proliferation in TNBC.

## 4. Discussion

In this section, we perform an analysis of the candidate miRNAs identified by the proposed feature selection method, using the available literature in cancer studies.

### 4.1. miRNAs from Cancer Type Classification

The five circulating miRNAs identified by our method as the most informative for cancer type classification are hsa-miR-122, hsa-let-7a, hsa-miR-23b, hsa-miR-708 and hsa-miR-200c.

hsa-miR-122 is a 22-nucleotide RNA molecule that plays an important role in liver functions [54]. It is related to regulation of cholesterol, fatty acid metabolism, and hepatocytes differentiation. Evidence indicates that hsa-miR-122 acts like a tumor suppressor, and its depletion is related to liver inflammation and hepatocellular cancer in mice [54,55]. In breast cancer, hsa-miR-122 has different expression patterns according to the subtype [56]. In addition, miR-122 promotes aggression and epithelial-mesenchymal transition in TNBC [57] and cell survival in radio-resistance cells [58]. High plasma miR-122 levels have been detected in AFP-producing gastric cancer [59].

The let-7 miRNAs show a high evolutionary conservation between organisms. Vertebrates have multiple let-7 isoforms and play an important role in development and tumor suppression [60]. hsa-let-7a is a member of the family and shows a downregulated expression in many tumor types like breast cancer [61,62], lung adenocarcinoma [63] and gastric cancer [64].

hsa-miR-23b is known to target tumor suppressor and cancer promoter genes. hsa-miR-23b is dis-regulated in proliferation, invasion, migration, apoptosis, autophagy and cell survival [65]. As a circulating biomarker, hsa-miR-23b is downregulated in colon cancer measured in plasma [66]. In contrast, it is upregulated in gastric cancer [67], lung cancer [68] and pancreatic cancer [69].

hsa-miR-708, also known as miR-708-5p, is a microRNA encoded within an intron of the ODZ4 gene. It can be found in different tissues with varying expression patterns like reproductive, secretory, muscle, gastrointestinal, nervous and lung [70]. hsa-miR-708 acts as a tumor suppressor or oncogene according to the cancer type. It has been associated with poor prognosis in lung adenocarcinoma [71] and carcinogenesis in colon [72] and bladder [73]. On the other hand, normal levels of hsa-miR-708 decrease cell growth and invasion and increase apoptosis in renal cancer cells [74].

hsa-miR-200c has been identified in lung, gastric, breast, ovarian and endometrial cancer with different expression patterns related to prognosis, aggressiveness and chemoresistance [75,76]. Moreover, hsa-miR-200c is involved in signaling cascades such as TGF-β, PI3K/Akt, Notch, VEGF, and NF-κB making it a candidate biomarker in cancer [77].

The result with the smallest *p*-value from the enrichment analysis in the KEGG dataset identified a strong relationship between the P53 signaling pathway and hsa-miR-122, hsa-let-7a, hsa-miR-23b, hsa-miR-708 and hsa-miR-200c. P53 is an important tumor suppressor that regulates the expression of many genes and is one of the most common mutated genes in cancer. Many miRNAs work as direct and indirect mediators of the P53 activity and the components of its pathway [78,79]. Moreover, the normal function of this tumor suppressor helps the maturation of some miRNAs with growth-suppressing function [80].

On the other hand, the first result in the enrichment analysis in the GO:BP dataset was cellular stress response. In normal cells, there is a balance between the activation of survival and cell death pathways, according to the type and duration of stress [81]. Cancer cells develop molecular mechanisms that facilitate their adaptation to different conditions like oxidative, metabolic, mechanical and genotoxic stresses, avoiding the restriction of the growth and increasing cell proliferation [82]. Importantly, miRNAs have the capacity to modify the stress response in cancer by making cells more susceptible or resistant to chemotherapy [83]. These findings prove that miRNAs play an important role in cancer biology and could be used as powerful circulating biomarkers for diagnosis and prognosis in human malignancies.

### 4.2. miRNAs from Triple-Negative Breast Cancer Classification

From our analysis, we selected 5 candidate miRNAs that are the most informative to separate cancer TNBC from the other subtypes in BRCA: hsa-miR-378*, hsa-miR-221, hsa-miR-342-3p, hsa-miR-630 and hsa-miR-145. All of them had already been shown to have potential as circulating cancer biomarkers in cancer studies, e.g., [84,85,86,87,88,89,90,91,92].

hsa-miR-378* is considered an onco-miRNA for its relationship with tumor growth and cell renewal. It is associated with the progression of breast cancer and the Warburg effect. Furthermore, hsa-miR-378* is capable of discriminating between breast cancer patients and controls [84,85].

Evidence indicates that hsa-miR-221 is upregulated and that its expression is related to proliferative pathways [93,94]. Several studies have linked the microRNA cluster 221/222 with chemoresistance. The miR-221/222 expression participates in the clinically aggressive basal-like subtype [95] and tamoxifen resistance in ER-positive breast cancer cells [87,88]. Furthermore, this cluster interfers with ERα expression [87] and miR-221/222 knockdown induces growth arrest and apoptosis in cells exposed to tamoxifen [88].

On the other hand, hsa-miR-342-3p expression correlates with ERα mRNA expression and its downregulation is related to tamoxifen resistance. hsa-miR-342-3p plays an important role in the therapy response of tamoxifen in ER-positive breast cancer [86,89]. Moreover, hsa-miR-342-3p activity affects some metabolic pathways like lactate and glucose fluxes in TNBC [35].

hsa-miR-630 is considerably suppressed in BRCA [90]. From in vitro experiments in which hsa-miR-630 mimics was transfected into MDA-MB-231 cells, it could be detected that the expression of hsa-miR-630 was decreased. miR-630 was also capable in inhibiting MDA-MB-231 cell migration and invasion targeting SOX4-3’-UT. Additionally, the SOX4 overexpression plasmid was transfected to further confirm that hsa-miR-630 played its role by downregulation [96].

Finally, hsa-miR-145 acts as a tumor suppressor through the inhibition of different proteins like ERBB3 and RTKN [91,92]. Additionally, hsa-miR-145 cooperates with P53 and has a proapoptotic effect in patients with breast cancer [97].

The miRNet enrichment analysis yields that P53 and the negative regulation of cell proliferation were the signaling pathways mostly involved with these miRNAs. Furthermore, the MTDH, IGF1R and CDK6 genes are targeted by at least 3 of the 5 miRNAs used to identify TNBC. Zare et al. [98] described the interplay of methilation patterns in miRNAs and the epithelial-mesenchymal transition. They identified that some genes like MTDH, IGF1R and CDK6 can be affected by miRNAs and can modify cellular processes in breast cancer.

## 5. Conclusions

miRNAs are known to play important roles in cellular biology processes such as differentiation, proliferation and apoptosis. Several research lines suggest that miRNAs are involved in different aspects of cancer, and recent studies indicate that there is potential in using their expression profiles as molecular signatures in clinically relevant settings.

miRBase (v22.1) consists of 1917 stem-loop sequences and 2657 mature sequences for human miRNAs [99]. Only some of these 2657 mature sequences are circulating miRNAs, and from that quantity, only 253 can be measured in blood, urine, plasma, serum or saliva (excluding pancreatic juice and cerebrospinal fluid). In this paper, our aim has been to reduce as much as possible the number of miRNAs necessary to classify cancer tumor types and to identify TNBC in BRCA. Our proposed approach consists in applying a recursive ensemble feature selection algorithm to reduce the original 253 miRNAs to 5 for each case study considered while, at the same time, ensuring high-quality classification (>90% mean classification accuracy over all the ensemble). It is important to state that our results are based on readily available clinical studies from the GEO repository.

Using the identified 5-miRNA signature for tumor classification, the classifier random forest obtains a mean accuracy of 97.61% in a 10-fold cross-validation, providing both results of high quality and a compact, human-interpretable list of miRNAs. When compared to the state-of-the-art in feature selection, our methodology was proven to be better than GALGO and ensemble-based approaches with an homogeneous topology, with a significant statistical difference ($p<10^{-4}$ using a standard Welch’s T-test). In the TNBC example, the signature obtained by our methodology outperforms the 31-miRNA signature from [35]. These remarkable results stem from the use of machine learning algorithms which are able to consider the influence of groups of features (in this case miRNAs) at the same time, while previous works only employed univariate statistics. Such an outcome is consistent with Mootha et al. [100], which makes the case for considering gene sets instead of individual genes. This methodology can be applied in other problems, such as differentiating between tumors with and without metastasis (Appendix C), and it is not restricted to only miRNAs but can also be used in mRNA data. In contrast to other methods such as Saha et al. [20], it is not limited by the number of variables (Appendix D).

This analysis is a first step towards assembling new approaches for cancer detection using circulating miRNAs, as measuring only 5 miRNAs levels is not only easier but also more resistant to measurement errors than to try and measure all available miRNAs levels. This research line might help the development of new concepts for prevention, secondary prevention and novel therapies.

## Figures and Tables

**Table 1:** Gene expression omnibus (GEO) repository datasets of miRNA cancer studies used in the project for platforms GPL8786, GPL14613 and GPL16384. BRCA: breast cancer; ESCA: esophageal cancer; HSNC: head and neck squamous cell cancer; LIHC: liver hepatocellular cancer; PRCA: prostate cancer; GBM: gliobastoma; CRC: colorectal cancer; NSCLC: non-small-cell lung cancer; GC: gastric cancer; OVC: ovarian cancer.

Dataset	Samples	Type	Reference	Class	Platform
GSE48088	33	BRCA	[34]	0	GPL14613
GSE86277	72	BRCA	[35]	0	GPL14613
GSE86278	49	BRCA	[35]	0	GPL14613
GSE86281	50	BRCA	[35]	0	GPL16384
GSE55856	108	ESCA	[36]	1	GPL14613
GSE34496	44	HSNC	-	2	GPL8786
GSE67138	57	LIHC	-	3	GPL8786
GSE67139	115	LIHC	-	3	GPL8786
GSE116182	64	LIHC	-	3	GPL14613
GSE36802	21	PRCA	[37]	4	GPL8786
GSE45604	50	PRCA	[38]	4	GPL14613
GSE104554	38	GBM	[39]	5	GPL14613
GSE110402	75	CRC	[40]	6	GPL14613
GSE46729	24	NSCLC	-	7	GPL8786
GSE63121	15	GC	[41]	8	GPL8786
GSE47841	30	OVC	[42]	9	GPL14613

**Table 2:** Comparison of the results of the different feature selection algorithms, reduced from the initial 253 to 5 features to differentiate cancer types.

	Heterogeneous		Homogeneous		GALGO			
	Ens. 5 Feats.		Ens. 5 Feats.		5 Feats.		253 Feats.	
	μ	σ	μ	σ	μ	σ	μ	σ
Gradient Boosting	0.9751	0.0134	0.9797	0.0154	0.8374	0.0453	0.975	0.0128
Random Forest	0.9761	0.0192	0.9854	0.0155	0.8656	0.0383	1	0
Logistic Regression	0.8877	0.0239	0.8777	0.0281	0.4954	0.0416	1	0
Passive Aggressive	0.8239	0.0544	0.8144	0.0707	0.4545	0.0590	1	0
SGD	0.8937	0.0305	0.8632	0.0362	0.5204	0.0832	0.9941	0.0078
SVC	0.9620	0.0197	0.9499	0.0186	0.5308	0.0454	1	0
Ridge	0.8083	0.0272	0.6900	0.0173	0.5010	0.0451	0.9977	0.0045
Bagging	0.9702	0.0193	0.9643	0.0165	0.8418	0.0425	0.9894	0.0121
Global	0.9121	0.0260	0.8906	0.0273	0.6309	0.0500	0.9945	0.0047

**Table 3:** Accuracy on the 10% data for testing the feature selection algorithm: Results for the signatures found by the heterogenous recursive ensemble feature selection algorithm, the homogeneous recursive ensemble feature selection algorithm, K-Best feature selection algorithm using f-score as selection citeria, 3 random feature subsets and a random shuffle of the test labels.

	Heterogeneous	Homogeneous	Univariate	GALGO	Random 1	Random 2	Random 3	Shuffle
Gradient Boosting (n_estimators = 300)	0.9412	0.9294	0.9412	0.9176	0.8824	0.8353	0.8000	0.2471
Random Forest (n_estimators = 300)	0.9412	0.9529	0.9412	0.9059	0.8941	0.8235	0.8235	0.2471
Logistic Regression	0.9059	0.8824	0.8588	0.8706	0.6353	0.5412	0.5882	0.2824
Passive Aggressive	0.8706	0.7765	0.7176	0.8471	0.4235	0.4118	0.5294	0.1765
SGD	0.8824	0.8588	0.7765	0.7882	0.5294	0.4235	0.3765	0.2353
SVC(linear)	0.9765	0.9176	0.8941	0.8588	0.6235	0.6235	0.5412	0.2824
Ridge	0.8118	0.7059	0.7412	0.7059	0.5882	0.4588	0.4000	0.2706
Bagging (n_estimators = 300)	0.9412	0.9294	0.9176	0.8824	0.8706	0.8471	0.8235	0.2118
Average	0.9089	0.8691	0.8485	0.8471	0.6809	0.6206	0.6103	0.2442

**Table 4:** Matthews correlation coefficient values for the 10% data left for testing the feature selection algorithm: The results for the heterogenous recursive ensemble feature selection algorithm, the homogeneous recursive ensemble feature selection algorithm, K-Best feature selection algorithm using f-score as selection citeria, 3 random feature subsets and a random shuffle of the test labels.

	Heterogeneous	Homogeneous	Univariate	GALGO	Random 1	Random 2	Random 3	Shuffle
Gradient Boosting (n_estimators = 300)	0.9346	0.9216	0.9346	0.9085	0.8693	0.8170	0.7778	0.1634
Random Forest (n_estimators = 300)	0.9346	0.9477	0.9346	0.8954	0.8824	0.8039	0.8039	0.1634
Logistic Regression	0.8954	0.8693	0.8431	0.8562	0.5948	0.4902	0.5425	0.2026
Passive Aggressive	0.8562	0.7516	0.6863	0.8301	0.3595	0.3464	0.4771	0.0850
SGD	0.8693	0.8431	0.7516	0.7647	0.4771	0.3595	0.3072	0.1503
SVC(linear)	0.9739	0.9085	0.8824	0.8431	0.5817	0.5817	0.4902	0.2026
Ridge	0.7908	0.6732	0.7124	0.6732	0.5425	0.3987	0.3333	0.1895
Bagging (n_estimators = 300)	0.9346	0.9216	0.9085	0.8693	0.8562	0.8301	0.8039	0.1242
Average	0.8987	0.8546	0.8317	0.8301	0.6454	0.5784	0.5670	0.1601

**Table 5:** μ and σ for accuracy and MCC over 10 runs using the top 5 features, for each algorithm.

	Accuracy		MCC	
	μ	σ	μ	σ
Heterogeneous	0.8840	0.0120	0.8691	0.0156
Homogeneous	0.8518	0.0183	0.8353	0.0204
GALGO	0.8227	0.0255	0.8132	0.0338

**Table 6:** Top 10 miRNet enrichment analyses in the Kyoto Encyclopedia of Genes and Genomes (KEGG) dataset for miRNAs hsa-miR-122, hsa-let-7a, hsa-miR-23b, hsa-miR-708 and hsa-miR-200c.

Pathway	Total	Expected	Hits	Pval
p53 signaling pathway	68	1	10	3.70 × 10${}^{-6}$
Pathways in cancer	310	4.57	19	3.70 × 10${}^{-6}$
Prostate cancer	87	1.28	11	3.70 × 10${}^{-6}$
Glioma	65	0.958	8	0.000207
Melanoma	68	1	7	0.00196
Bladder cancer	29	0.428	5	0.00196
Colorectal cancer	49	0.722	6	0.00217
Chronic myeloid leukemia	73	1.08	7	0.00227
Focal adhesion	200	2.95	11	0.00327
Small cell lung cancer	80	1.18	7	0.00327

**Table 7:** Top 10 miRNet enrichment analyses in the Gene Ontology-Biological Process (GO:BP) dataset for miRNAs hsa-miR-122, hsa-let-7a, hsa-miR-23b, hsa-miR-708 and hsa-miR-200c.

Pathway	Total	Expected	Hits	Pval
Cellular response to stress	1620	15.4	44	3.03 × 10${}^{-8}$
Positive regulation of cell proliferation	786	7.43	27	1.66 × 10${}^{-6}$
Response to hypoxia	245	2.31	15	2.53 × 10${}^{-6}$
Regulation of cell cycle	886	8.37	28	2.53 × 10${}^{-6}$
Regulation of cell proliferation	1430	13.5	36	4.30 × 10${}^{-6}$
Response to abiotic stimulus	876	8.28	27	5.44 × 10${}^{-6}$
Negative regulation of cell cycle	520	4.91	20	1.04 × 10${}^{-5}$
Regulation of molecular function	2250	21.2	46	1.08 × 10${}^{-5}$
Regulation of cyclin-dependent protein kinase activity	89	0.841	9	1.40 × 10${}^{-5}$
Negative regulation of apoptotic process	679	6.42	22	2.56 × 10${}^{-5}$

**Table 8:** Accuracy comparison for all classifiers, using all 253 features, the 5-miRNA signature found by the proposed approach, and the 31-miRNA signature from Romero et al. for separating triple-negative from the rest of the BRCA subtypes.

	5 Feats.		253 Feats.		Romero et al.	(31 Feats.)
**Classifier**	μ	σ	μ	σ	μ	σ
GradientBoosting	0.9345	0.0523	0.9134	0.0487	0.9239	0.0485
RandomForest	0.9354	0.0617	0.9459	0.0416	0.9184	0.0432
LogisticRegression	0.9243	0.0487	0.9406	0.0612	0.8958	0.0643
PassiveAggressive	0.9076	0.0550	0.9076	0.0778	0.8797	0.0637
SGDClassifier	0.9085	0.0628	0.8918	0.0770	0.8692	0.0700
SVC(linear)	0.9243	0.0487	0.9242	0.0655	0.8572	0.0400
Ridge	0.9079	0.0754	0.9085	0.0533	0.8856	0.0611
Bagging	0.9295	0.0411	0.9076	0.0544	0.9341	0.0412
Global	0.9215	0.0557	0.9175	0.0599	0.8955	0.0540

**Table 9:** Top 10 miRNet enrichment analysis results for miRNAs hsa-miR-378*, hsa-miR-221, hsa-miR-342-3p, hsa-miR-630 and hsa-miR-145 using the KEGG database.

Pathway	Total	Expected	Hits	Pval
p53 signaling pathway	68	0.509	6	0.000518
Pancreatic cancer	69	0.516	6	0.000518
Glioma	65	0.486	6	0.000518
Melanoma	68	0.509	6	0.000518
Chronic myeloid leukemia	73	0.546	6	0.000576
Bladder cancer	29	0.217	4	0.00197
Cell cycle	124	0.927	6	0.00821
Pathways in cancer	310	2.32	9	0.009
Non-small cell lung cancer	52	0.389	4	0.0133
Adherens junction	70	0.524	4	0.0368

**Table 10:** Top 10 miRNet enrichment analysis results for miRNAs hsa-miR-378*, hsa-miR-221, hsa-miR-342-3p, hsa-miR-630 and hsa-miR-145 using the GO:BP database.

Pathway	Total	Expected	Hits	Pval
negative regulation of cell proliferation	585	2.7	12	0.00631
regulation of cell proliferation	1430	6.6	19	0.00631
cell proliferation	1900	8.79	22	0.00674
G1 phase of mitotic cell cycle	47	0.217	4	0.00882
enzyme linked receptor protein signaling pathway	1180	5.43	16	0.00882
myeloid cell differentiation	296	1.37	8	0.00882
G1 phase	49	0.226	4	0.00882
response to endogenous stimulus	1360	6.3	17	0.0114
positive regulation of cell proliferation	786	3.63	12	0.0166
response to organic substance	2500	11.5	24	0.0166

**Figure A1:**
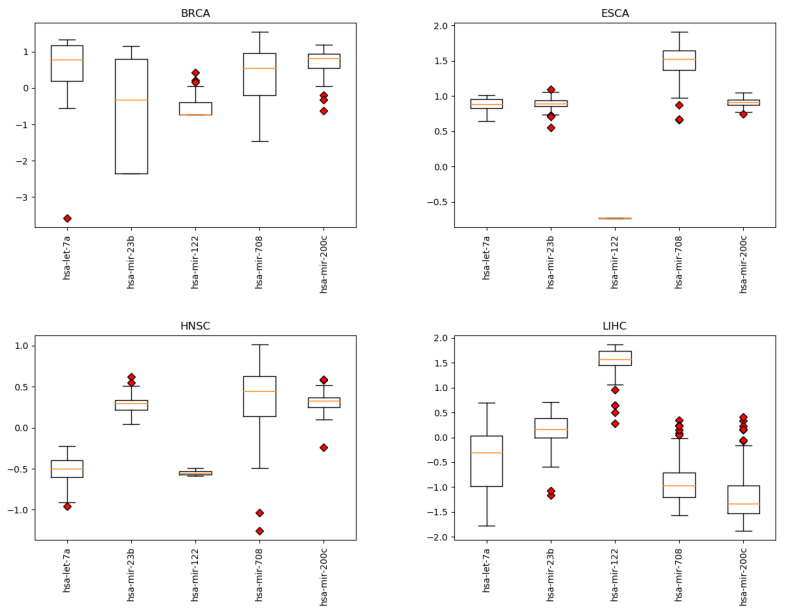

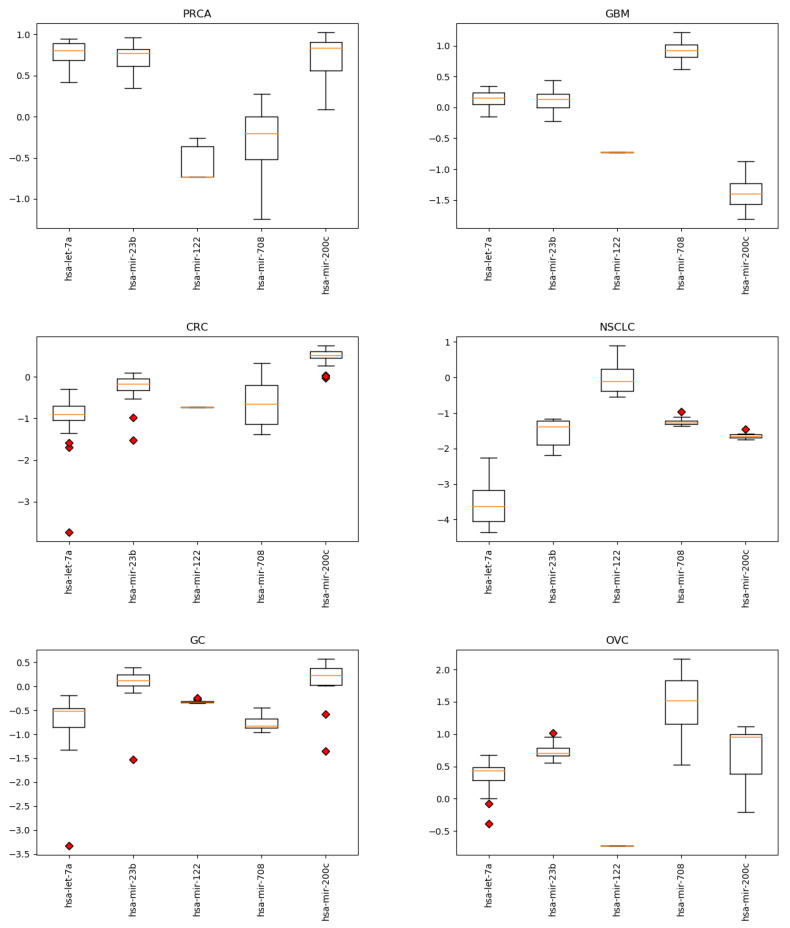
Expression levels by cancer type for the 5 miRNAs identified by the proposed approach. Cancer types: breat (BRCA); esophageal (ESCA); head and neck squamous cell (HNSC); liver hepatocelluar (LIHC); prostate (PRCA); gliobastoma (GBM); colorectal (CRC); non-small-cell lung (NSCLC); gastric (GC); ovarian (OVC).

**Figure A2:**
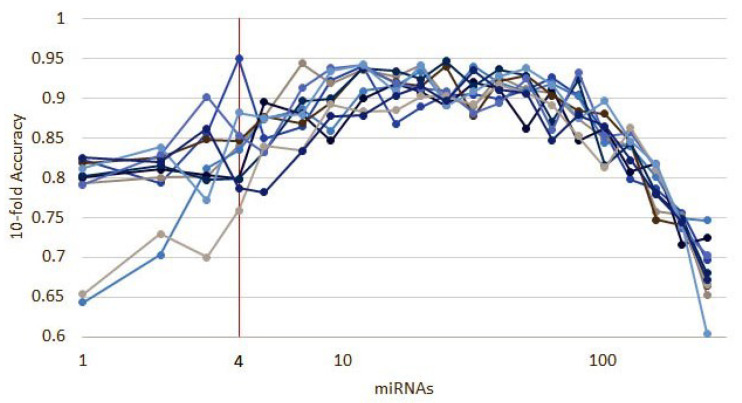
Ten runs of the heterogeneous ensemble recursive selection algorithm: From the 10 runs, the highest accuracy is given by 4 miRNAs: hsa-mir-486-3p, hsa-mir-21, hsa-mir-1285, hsa-miR-708 and hsa-mir-638.

**Figure A3:**
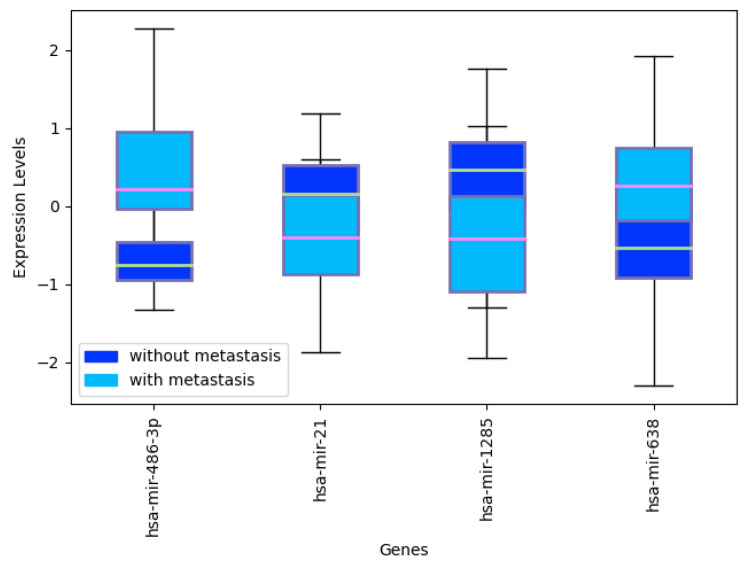
Expression levels of selectedmiRNAs: hsa-mir-486-3p, hsa-mir-21, hsa-mir-1285, hsa-miR-708 and hsa-mir-638 in CRC samples with and without metastasis.

**Figure A4:**
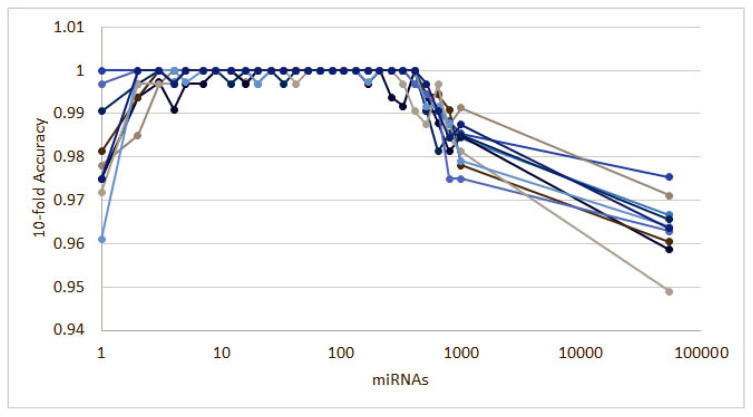
Ten runs of the heterogeneous ensemble recursive selection algorithm: From the 10 runs, the highest accuracy is given by MUC4 gene expression alone.

**Figure A5:**
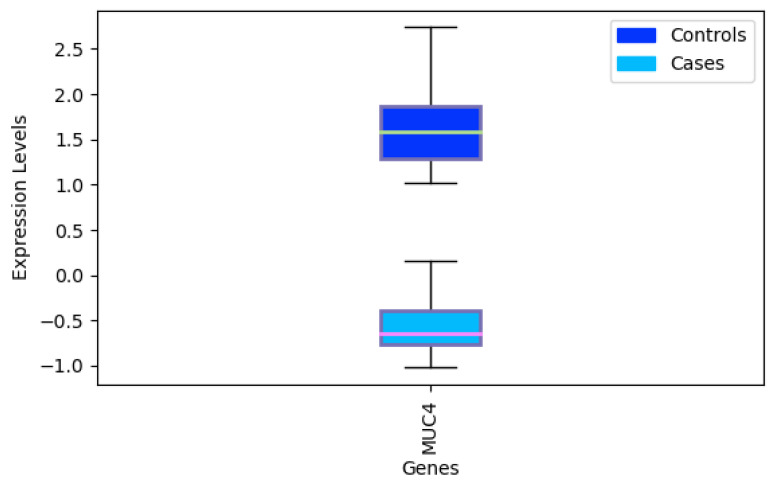
Expression levels of MUC4 and difference between tumor tissue and controls.

## Sample Availability

The datasets generated/analyzed for this study can be found in the GitHub repository https://github.com/steppenwolf0/circulating.

## Appendix A. Circulating miRNAs

In Table A1, we present the list of all 253 circulating miRNAs identified in the dataset, using an analysis of the available literature.

**Table A1:** List of all circulating miRNAs.

let-7a	miR-140-3p	miR-19b	miR-335	miR-513a-3p
let-7a*	miR-141	miR-200a	miR-338-3p	miR-516b
let-7b	miR-142-3p	miR-200b	miR-338-5p	miR-518b
let-7c	miR-143	miR-200c	miR-339-3p	miR-520a-3p
let-7d	miR-144	miR-202	miR-339-5p	miR-548b-5p
let-7e	miR-145	miR-203	miR-340*	miR-557
let-7f	miR-146a	miR-205	miR-342-3p	miR-564
let-7g	miR-146b-3p	miR-206	miR-345	miR-566
let-7i	miR-146b-5p	miR-20a	miR-346	miR-571
miR-1	miR-148a	miR-20b	miR-34a	miR-574-3p
miR-100	miR-148b	miR-21	miR-34b	miR-574-5p
miR-101	miR-150	miR-210	miR-361-3p	miR-587
miR-106b	miR-150*	miR-212	miR-365	miR-589
miR-107	miR-151-5p	miR-214	miR-371-5p	miR-595
miR-10a	miR-152	miR-215	miR-372	miR-601
miR-10b	miR-155	miR-218	miR-373	miR-616*
miR-1182	miR-15a	miR-22	miR-375	miR-618
miR-122	miR-15b	miR-221	miR-376a	miR-622
miR-122*	miR-15b*	miR-222	miR-376c	miR-625
miR-1224-5p	miR-16	miR-223	miR-377	miR-625*
miR-1229	miR-16-2*	miR-23a	miR-378	miR-628-3p
miR-1231	miR-17	miR-23b	miR-378*	miR-629
miR-1245	miR-181a	miR-24	miR-379	miR-630
miR-1246	miR-181a-2*	miR-25	miR-382	miR-638
miR-1254	miR-181b	miR-26a	miR-409-3p	miR-646
miR-125b	miR-181d	miR-26b	miR-409-5p	miR-650
miR-125b-2*	miR-182	miR-27a	miR-410	miR-652
miR-126	miR-1825	miR-27b	miR-411	miR-654-3p
miR-1260	miR-183	miR-296-5p	miR-421	miR-656
miR-1268	miR-184	miR-298	miR-423-5p	miR-668
miR-127-3p	miR-185	miR-299-5p	miR-425	miR-675
miR-1275	miR-186	miR-29a	miR-425*	miR-7
miR-128	miR-187	miR-29b	miR-429	miR-708
miR-1280	miR-187*	miR-29c	miR-431	miR-744
miR-1284	miR-18a	miR-301a	miR-431*	miR-744*
miR-1285	miR-18b	miR-302b	miR-432	miR-760
miR-1288	miR-18b*	miR-30a	miR-451	miR-874
miR-1290	miR-190b	miR-30b	miR-452	miR-885-5p
miR-1295	miR-191	miR-30c	miR-454	miR-922
miR-129-5p	miR-192	miR-30c-1*	miR-454*	miR-92a
miR-1304	miR-193a-3p	miR-30d	miR-483-3p	miR-92a-2*
miR-130a*	miR-193b	miR-30e	miR-483-5p	miR-92b
miR-130b	miR-194	miR-31	miR-484	miR-93
miR-1323	miR-195	miR-32	miR-486-3p	miR-93*
miR-133a	miR-196a	miR-320a	miR-486-5p	miR-936
miR-133b	miR-196b	miR-320c	miR-487b	miR-939
miR-134	miR-197	miR-320d	miR-493	miR-942
miR-138	miR-198	miR-324-3p	miR-494	miR-99a
miR-138-2*	miR-199a-3p	miR-326	miR-497	miR-99b
miR-139-3p	miR-199a-5p	miR-328	miR-502-5p	
miR-139-5p	miR-19a	miR-331-3p	miR-504	

## Appendix C. miRNA Levels in CRC with and without Metastasis

To provide evidence that the proposed methodology can be used not only to classify tumors but also more in general to answer specific questions related to tumors, where miRNAs can be informative, we applied our algorithm to dataset GSE53159 [101], separating metastasized tumors from those which are not. This dataset is composed of 32 samples and 16 colorectal cancer (CRC) samples with liver metastasis and 16 CRC samples without liver metastasis for platform GPL8786. After applying our method, we obtain a 4-miRNA signature, with the differentially expressed hsa-mir-486-3p, hsa-mir-21, hsa-mir-1285, hsa-miR-708 and hsa-mir-638. The final average accuracy is 0.9312, with 0.8625 MCC.

## Appendix D. Recursive Ensemble Feature Selection in mRNA Data

While other techniques such as [20] can also be effective to identify signatures for bioinformatic applications, they are usually limited to working with a few hundreds of features. In order to show how our algorithm can be effective even with a large number of features, we apply it to dataset GSE12452 [102] that contains 54,675 features related to messenger RNA (mRNA). This dataset is composed of 41 samples: 31 samples are nasopharyngeal tumor tissue and 10 are normal nasopharyngeal healthy controls for platform GPL570. After applying our methodology, we obtain a signature composed of just one gene, MUC4, differentially expressed to separate tumor and healthy tissue. This is consistent with studies that point out MUC4 as a cancer biomarker [103,104]. Overall, the signature identified has a global accuracy of 1.0, with 1.0 MCC.
